# Application of citizen science with the nationwide bird census

**DOI:** 10.1038/s41598-024-61225-w

**Published:** 2024-05-06

**Authors:** Yerim Lee, Yuno Do, Maurice Lineman, Gea-Jae Joo, Hyunbin Jo

**Affiliations:** 1 Department of Integrated Biological Science, Pusan National University, Busan, Republic of Korea; 2 Department of Biological Sciences, Kongju National University, Gongju, Republic of Korea; 3 RCF Experimental School, Chaoyang District, Beijing, People’s Republic of China; 4 Korea Network for Rivers and Watersheds, Busan, Republic of Korea; 5 Department of Pet Health Care, Busan Health University, Busan, Republic of Korea

**Keywords:** Citizen science, Bird, Data quality, Reliability, National, Animal migration, Biodiversity

## Abstract

Citizen science (CS) is the most effective tool for overcoming the limitations of government and/or professional data collection. To compensate for quantitative limitations of the 'Winter Waterbird Census of Korea', we conducted a total of four bird monitoring via CS from 2021 to 2022. To use CS data alongside national data, we studied CS data quality and improvement utilizing (1) digit-based analysis using Benford’s law and (2) comparative analysis with national data. In addition, we performed bird community analysis using CS-specific data, demonstrating the necessity of CS. Neither CS nor the national data adhered to Benford's law. Alpha diversity (number of species and Shannon index) was lower, and total beta diversity was higher for the CS data than national data. Regarding the observed bird community, the number of species per family was similar; however, the number of individuals per family/species differed. We also identified the necessity of CS by confirming the possibility of predicting bird communities using CS-specific data. CS was influenced by various factors, including the perceptions of the survey participants and their level of experience. Therefore, conducting CS after systematic training can facilitate the collection of higher-quality data

## Introduction

As a top predator in ecosystem food chains, birds are one of the most sensitive ecosystem indicators affected by global environmental change (e.g. climate change and habitat fragmentation) because of their freedom of movement between habitats^1–4^. Therefore, studying bird communities is important to understand ecosystems and facilitate their conservation^5,6^. The Korean peninsula, located in the middle of the East Asian–Australasian Flyway (EAAF), provides important migratory bird habitats (such as wetlands, streams, lakes, reservoirs, and coasts), which are distributed nationwide^7,8^. These habitats serve as important stopovers for the wintering and breeding sites of migratory birds, particularly various waterbirds that breed in Siberia, Manchuria, and Mongolia^9^.

Due to the geographical features of the Korean Peninsula, the Ministry of Environment (MOE) of South Korea has been conducting a “Migratory Shorebird Monitoring” program to record migratory birds staging along the west and south coasts every spring and fall since 1993 and the “Winter Waterbird Census of Korea” to monitor birds arriving nationwide every winter since 1999^10,11^. These national data reports are important for understanding variation in the abundance of migratory bird communities within the EAAF, as well as for determining migratory bird distribution on the Korean Peninsula^11^. However, among the ongoing programs, nationwide monitoring is conducted only during the winter. In spring and fall, monitoring is limited to specific regions (southwest coast) and species (e.g. shorebirds). Furthermore, there is no monitoring program, even during the summer season^10–12^. Quantitative limitations commonly observed in governmental or professional monitoring arise from financial pressure and restrictions on the availability of expertise^13^. Therefore, another accompanying monitoring program is required to supplement the current government methods.

Citizen science (herein referred to as CS), in which the public participates in research to create new scientific knowledge, has proven to be a suitable tool in overcoming certain professional limitations^14^. Multiple benefits of CS have been noted. It helps to track large-scale environmental changes at a low cost^15^ and bridge gaps in data-scarce areas^16^. From another perspective, CS has many social benefits, including increased environmental democracy, citizen inclusion in local issues, scientific literacy, and social capital^15^. CS has been used in scientific fields such as astronomy and ecology, where large-scale data collection through observation has been important since the nineteenth century and has recently undergone further development owing to the growing accessibility of the Internet and smartphones^17,18^. CS offers numerous advantages and is widely used to gather extensive data across various fields^19^. Ornithology is a field with well-developed large-scale projects on a continental scale, such as the Christmas Bird Count (CBC), Breeding Bird Survey (BBS), and Project FeederWatch (PFW)^20^. National-scale projects have also been conducted in various countries, focusing on topics such as the monitoring of marine pollution, pests, and ecosystem services^21–23^. A data confirmation process is necessary to use citizen-collected data along with national data^24^. Although many CS studies include methods used in projects and how data should be validated, few of them have addressed the reliability of the dataset or compared it with national data^25,26^.

To compensate for the quantitative limitations of national monitoring, we conducted bird monitoring twice a year using the CS during winter and summer, starting in 2021. To use CS results alongside the national data, we studied data quality and improvement for CS. The goals of our study were to (1) identify the quality of CS and national data using Benford’s law, (2) find complementary points through comparative analysis with national data (herein referred to as Nation), and (3) suggest the necessity of using CS via the analysis of bird communities using related data. Furthermore, we discuss the improvements required in CS based on the derived research findings.

## Results

### Evaluation of the quality of CS and Nation Data based on Benford’s law

**Figure 1:**
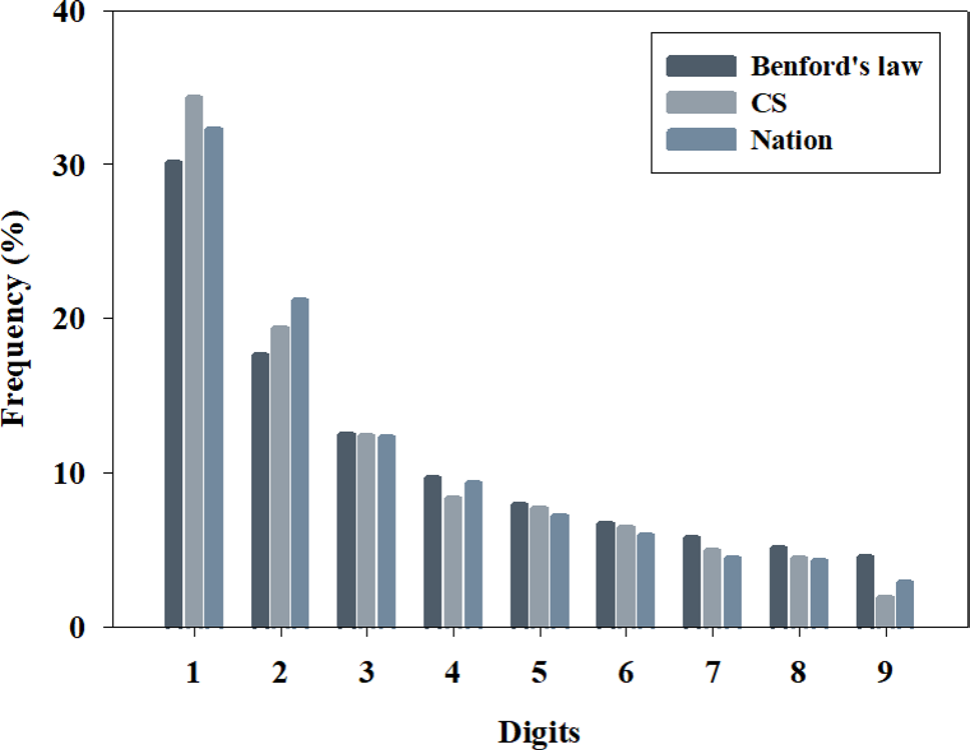
Comparison between frequency distributions of the first digit from citizen science (CS) and national data (Nation) with Benford’s law. Dark grey: Benford’s law; Grey: CS; Blue: Nation.

The frequency distributions calculated from the CS and Nation data were the highest for digit 1 and the lowest for digit 9, which corresponds to Benford’s law (Fig. 1). However, both frequency values differed from those expected based on Benford’s law. In the χ^2^ test, the null hypothesis—stating that both CS and Nation data adhered to Benford’s law––was rejected at a 99% confidence level. For both CS and Nation data, compared with Benford’s law, quantities with 1 and 2 as the first digits were more common, but first digits of 3–9 were less common (Table 1). In particular, CS had a higher frequency for digit 1 and a lower frequency for digit 9 than Nation data, indicating a greater deviation from values suggested by Benford's law.

**Table 1:** Frequency table of the first digit from citizen science (CS) and national data (Nation) compared with Benford’s law. BL: Benford’s law.

Digits	BL	CS		Nation	
		Observed	Data set	Observed	Data set
1	30.10%	1060	34.38%	4248	32.25%
2	17.61%	596	19.33%	2797	21.23%
3	12.49%	383	12.42%	1620	12.30%
4	9.69%	257	8.34%	1236	9.38%
5	7.92%	238	7.72%	953	7.23%
6	6.70%	199	6.45%	790	6.00%
7	5.80%	153	4.96%	586	4.45%
8	5.12%	138	4.48%	564	4.28%
9	4.58%	59	1.91%	379	2.88%
Chi-Square		84.1476**		279.8184**	

**99% significantly different from Benford.

For both CS and Nation data, the χ^2^ calculated by dividing the survey period was more similar to Benford’s law (see Supplementary Table S1). In the case of CS, it was more similar for summer monitoring data (χ^2^ 25.3604^**^, 17.0215^*^) than winter monitoring data (χ^2^ 30.5801^**^, 33.2766^**^). In particular, the lowest chi-square value was obtained for the 2022 summer monitoring period (17.0215^*^).

### Comparative bird diversity between CS and Nation data

**Figure 2:**
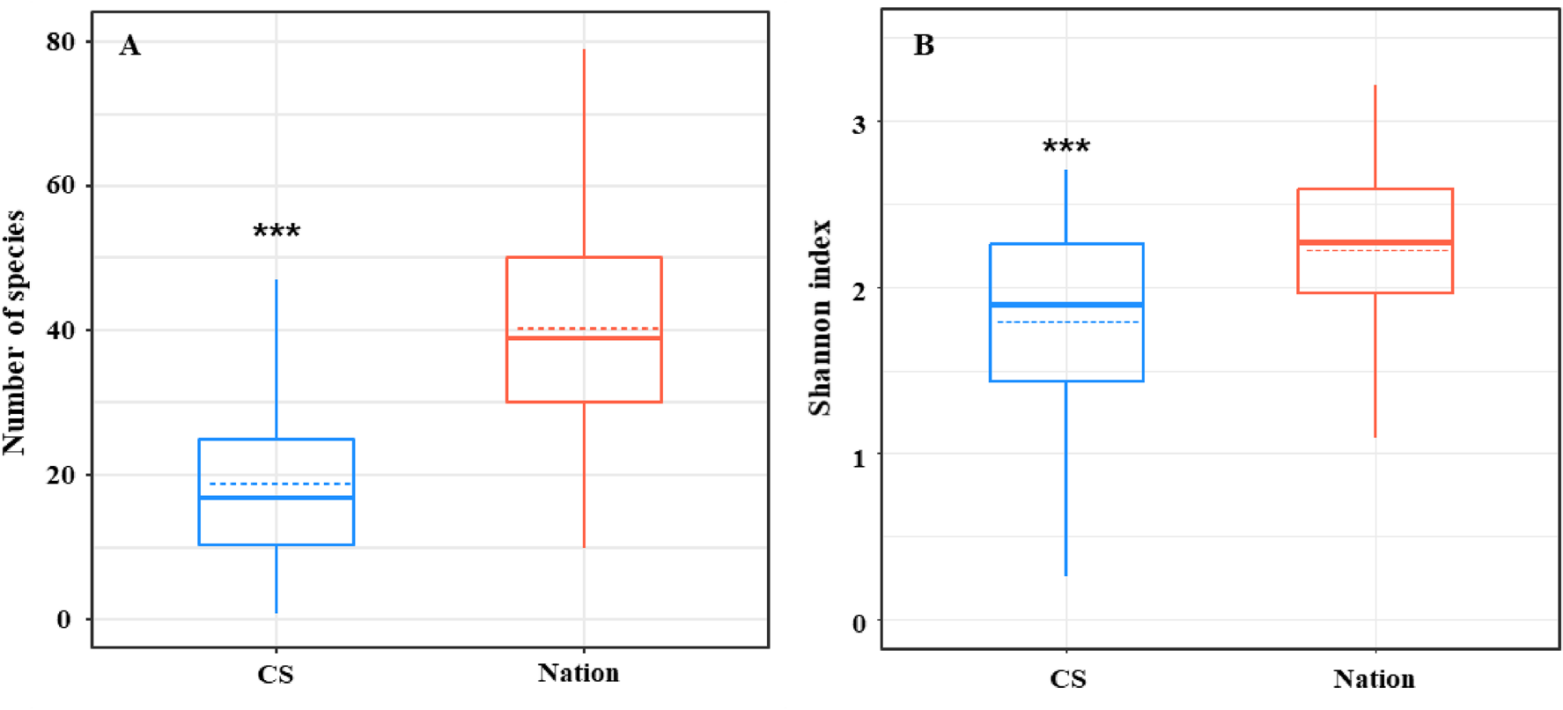
Alpha diversity of citizen science (CS) and national monitoring (Nation). (**a**) Difference in the number of species between the two methods (CS and Nation). (**b**) Shannon index comparing two methods. Box-and-whisker plots show the median (bold solid line) and mean (dashed line) values with first and third quartiles. ***p-value < 0.001.

**Figure 3:**
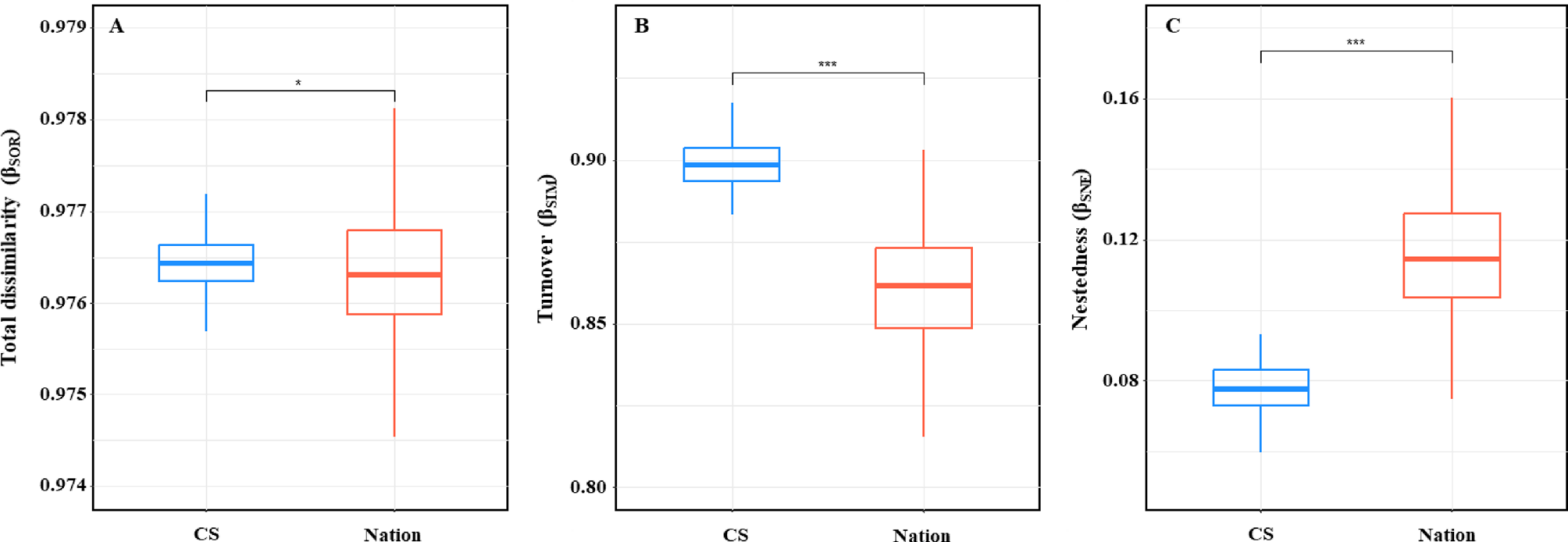
Beta diversity based on the Sørensen pairwise species dissimilarity in citizen science (CS) and national data (Nation). (**a**) Comparison of total dissimilarity (β_SOR_) between two methods (CS and Nation). (**b**) Difference in the turnover (β_SIM_) component of two methods. (**c**) Difference in the nestedness (β_SNE_) component of two methods. Box-and-whisker plots show the median (bold solid line) and mean (dashed line) values with first and third quartiles. *p-value < 0.05 and ***p-value < 0.001.

Species diversity within sites (alpha diversity) and species divergence between sites (beta diversity) were significantly different between CS and nation (Figs. 2 and 3). With respect to alpha diversity, CS data had a lower number of species (18.906 ± 1.012) and Shannon index (1.790 ± 0.608) than did Nation (number of species: 40.921 ± 0.793; Shannon index: 2.225 ± 0.523) (Mann–Whitney U test, p < 0.001).

In contrast to alpha diversity, total beta diversity (β_SOR_) was significantly higher for CS (0.9764 ± 0.0003) than for Nation data (0.9763 ± 0.0007) (t-test, p < 0.05) (Fig. 3). The results of the turnover (β_SIM_) and nestedness (β_SNE_) components were contrastive; β_SIM_ was higher for CS (0.8986 ± 0.0071) than for Nation data (0.8611 ± 0.0170), whereas β_SNE_ was higher for Nation data (0.1152 ± 0.0166) than for CS (0.0778 ± 0.0070) (t-test, p < 0.001). The turnover component of total beta diversity was considerably higher in both the CS and Nation data than the nestedness component (Fig. 3).

### Difference in bird communities at common sites for CS and Nation

**Figure 4:**
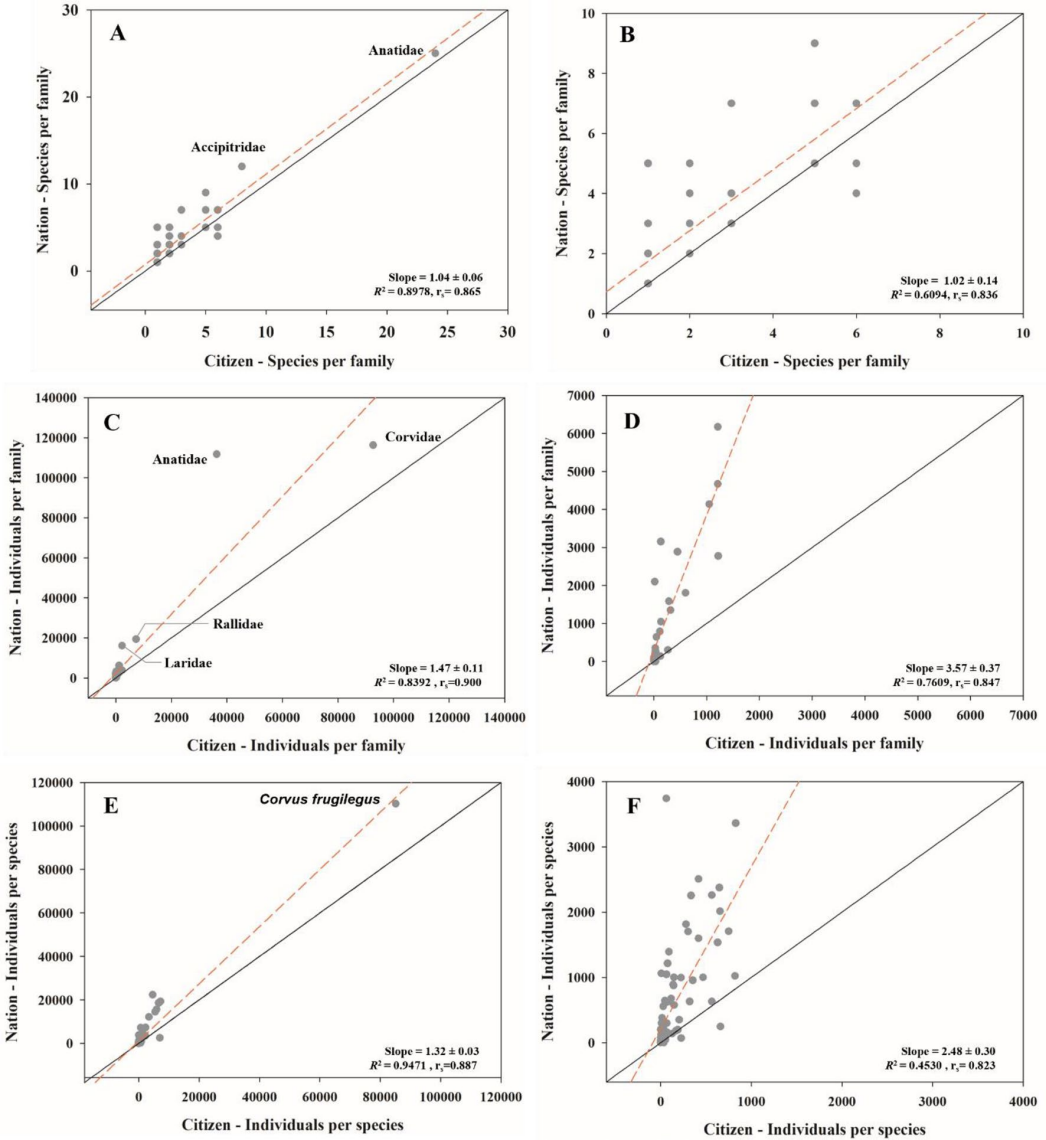
Scatterplot and linear regression line for species and individuals detected at 32 sites, surveyed by both methods (citizen science and national monitoring). (**a**) Number of species per family, (**c**) Number of individuals per family, (**e**) Number of individuals per species, (**b**, **d**, **f**) excluding outlier from (**a**, **c**, **e**). The solid black line indicates a 1:1 relationship between the values of the two methods. The actual line of regression between two method is indicated by the dashed red line. R^2^: Coefficient of determination of regression; r_s_: Correlation coefficient.

A total of 36 families were identified using both methods (CS and Nation). More than 10 Anatidae species were detected for both methods (Table 2). More than 10 species of Accipitridae were detected only in Nation data, and all other families showed fewer than 10 species for both methods. The correlation coefficient between Citizen and Nation data was 0.865 (p < 0.001), and the slope of the line was 1.04 ± 0.06. A slope value close to 1.0 indicated that the number of species per family observed by CS and Nation had the same tendency (Fig. 4a). When two families (Anatidae and Accipitridae) calculated as outliers were removed, the correlation coefficient was 0.836 (p < 0.001) and the slope was 1.02 ± 0.14, which was comparable to the value obtained prior to outlier removal (Fig. 4b).

**Table 2:** Number of species and individuals per family observed by two methods (citizen science and national data) in February 2021–2022. CS: citizen science; Nation: national data.

Family	CS		Nation	
	Species	Individuals	Species	Individuals
Phasianidae	1	14	1	77
Anatidae	24	36,354	25	111,711
Gaviidae	1	35	2	3
Podicipedidae	3	287	4	1585
Ciconiidae	1	1	1	2
Threskiornithidae	1	127	1	140
Ardeidae	6	604	5	1805
Phalacrocoracidae	2	2241	2	3811
Falconidae	2	22	2	63
Accipitridae	8	268	12	304
Rallidae	2	7315	3	19,320
Gruidae	3	1220	3	2774
Haematopodidae	1	2	1	38
Charadriidae	2	26	5	193
Scolopacidae	3	19	7	2097
Laridae	5	2268	9	16,080
Columbidae	2	1212	3	4671
Alcedinidae	1	4	1	4
Upupidae	1	6	1	45
Picidae	1	4	5	102
Laniidae	2	47	3	110
Corvidae	6	92,683	7	116,210
Paridae	2	113	4	791
Aegithalidae	1	32	1	278
Alaudidae	1	49	1	644
Pycnonotidae	1	138	2	1047
Timaliidae	1	1051	1	4140
Zosteropidae	1	2	1	78
Troglodytidae	1	3	1	43
Turdidae	2	30	3	356
Muscicapidae	1	32	3	247
Passeridae	1	1214	1	6174
Prunellidae	1	2	1	16
Motacillidae	6	319	4	1351
Fringillidae	5	450	5	2886
Emberizidae	5	133	7	3155
Totals	107	148,327	138	302,351

Corvidae had the greatest number of individuals per family for both methods, with more than 90,000 individuals, followed by Anatidae (Table 2). The correlation coefficient and slope of the line between CS and Nation were 0.900 (p < 0.001) and 1.47 ± 0.11, respectively (Fig. 4c). When five families (Anatidae, Phalacrocoracidae, Rallidae, Laridae, and Corvidae) were calculated as outliers, the correlation coefficient was 0.847 (p < 0.001), and the slope was 3.57 ± 0.37 (Fig. 4d). The correlation coefficient was similar to that obtained prior to outlier removal, whereas the slope increased by approximately 2.5 times, from 1.4 to 3.5. Thus, for each individual in a family recorded using CS, nearly 2.5 individuals of that family were recorded by the nation.

The dominant species for both methods was *Corvus frugilegus*. The two methods shared 8 of the top 10 species, but the ranking differed between the two methods. Of the top 20 species identified in each approach, 16 were shared; however, they had different ranks. The correlation coefficient is 0.887. The slope of the line was 1.32 ± 0.03 (close to 1.0), but when excluding the 15 calculated outlier species, the slope increased to 2.48 ± 0.30 (Fig. 4e,f). Thus, for every individual of each species recorded in the CS, nearly two individuals of that species were recorded in the Nation.

### Prediction of bird individuals by CS-specific data

River attributes and survey content were examined using multiple linear regression analyses to evaluate their effects on bird abundance (Table 3). Two variables showed a significant effect on bird abundance in winter (river attributes: R^2^ = 0.332, p < 0.001; survey content: R^2^ = 0.203, p < 0.01) but not in summer (river attributes: R^2^ = 0.133, p = 0.062; survey content: R^2^ = 0.009, p = 0.793).

**Table 3:** Multiple linear regression of citizen science-specific data on individuals. Only winter data (February, 2021–2022) for analysis. B: Unstandardised regression coefficient; SE: Standard Error; Beta: Standardised coefficients; R^2^: Coefficient of determination; ∆R^2^: Adjusted R^2^.

	B	SE	Beta	R^2^	ΔR^2^
River attributes					
Model				0.332	0.288
Constant	4.144	0.616			
Width of riparian	0.022	0.272	0.019		
Width of channel	0.691	0.269	0.596*		
Water depth	− 0.657	0.429	− 0.173		
Freezing rate	0.075	0.088	0.090		
Survey contents					
Model				0.203	0.178
Constant	4.477	0.852			
Number of citizens	− 0.200	0.339	− 0.067		
Distance	1.266	0.329	0.437***		

***p < 0.001, *p < 0.05.

Of the four river attributes measured in winter, only the width of channel had a significant positive effect on bird abundance (B = 0.691, p = 0.013). Only distance had a significant positive effect on bird abundance based on both survey contents (B = 1.266, p < 0.001).

## Discussion

We found that data collected from citizens and professionals (nationals) did not adhere to Benford's law. In both datasets, a higher frequency of digit 1 and a lower frequency of digit 9 was noted compared with Benford’s law. Wintering migratory birds form large flocks^27^. Accurate counting of the number of birds in a large flock is challenging; therefore, observers usually use the technique of visually breaking the flock into groups of 10, 100, or 1000, and then estimate the number of units within the flock^28,29^. Benford's law may not hold for measurements in which random numbers have been applied, or where human intervention has occurred^30,31^. The bird numbers counted as an estimate fall into the latter category; further, it appears that they do not adhere to Benford's law. Benford's law has primarily been employed in economics and social sciences to detect fraud in accounting or tax data. Its applicability has recently been confirmed in the natural sciences (e.g. physics, astronomy, geophysics), but its use is not yet active^32,33^. In future research, it will be necessary to identify the accuracy of the data through the re-established Benford's law or new techniques, considering the characteristics of data recorded as estimates for count numbers.

We identified differences in species diversity between the CS and Nation data. For alpha diversity, both the number of species and Shannon index were higher for the Nation obtained by professionals, whereas total beta diversity, which indicates the extent of change in species composition among spaces, was higher for CS. Alpha diversity differences can arise in two ways: (1) professionals and citizens may differ in their ability to detect or count species^19^. Professionals are less affected by difficulties in detection or identification because of their accumulated experience and knowledge, whereas citizens with less experience and knowledge are more affected^24,34^. Species that are difficult to detect or count (e.g. lesser-known species and species inhabiting large flocks) are less likely to be observed or identified by citizens, which explains why the alpha diversity is lower^35,36^. (2) Monitoring sites for professionals and citizens may exhibit different characteristics. Unlike Nation, where sites were evenly distributed across the country and monitored across various topographies, in this study, sites were selected by the volunteers and thus concentrated in urban areas near the Han, Nakdong, and Yeongsan rivers). In urban areas with high population density, the area near the river is maintained as a park, allowing people to access the riverside land, and because of the low biodiversity, including vegetation, the number of birds detected—alpha diversity—is less than that of non-urban areas^37–39^.

With beta diversity, both total dissimilarity (β_SOR_) and turnover component (β_SIM_) were higher in CS, whereas nestedness component (β_SNE_) was higher in Nation data. In particular, turnover was significantly higher than nestedness in both cases (approximately 11 times in CS and 7 times in Nation data), indicating that turnover contributed much more to the total beta diversity. This implies that turnover measures largely the same phenomenon as does total dissimilarity^40^. Turnover, which indicates the replacement of some species with others, is lower when the number of shared species between sites is high^41^. Larger sample areas tend to have more diverse assemblages, indicating greater species overlap between sites^42^. Regarding distance, the number of shared species tended to be higher when the distance was shorter and lower when the distance was greater^43,44^. Nation sites include the main river channels or coastal areas, which have larger sample areas, whereas CS focuses on small portions of urban rivers or wetlands, resulting in smaller sample areas. Furthermore, Nation sites are evenly distributed across the country, resulting in shorter distances between sites. However, CS sites are concentrated around urban areas near major rivers such as the Han, Yeongsan, and Nakdong Rivers, resulting in longer distances between sites. Therefore, the difference in total beta diversity between CS and Nation data can be attributed to the turnover component resulting from variations in the size and distance between the sites.

Differences were observed not only in diversity but also in the actual measured values (the number of species and individuals) between the Nation and CS data. We found that the number of species per family was detected at a similar level in CS and Nation, but the number of individuals per family and species was detected more frequently in the Nation (Fig. 4). However, the species with high detection rates exhibited a high degree of similarity. Furthermore, birds that have been frequently exposed to the public, such as *C. frugilegus* (Fig. 4e), which has been reported in news articles to form large flocks in winter, and Anatidae (Fig. 4c), which have a close relationship with human culture based on their domestication history, were comparably detected by both citizens and professionals at a similar level^45–47^. These findings indicate the significance of raising public awareness as a key element in enhancing the reliability of CS.

Despite the current limitations in the reliability of the data collected by citizens, we identified the rationale for the necessity of CS while operating independently from national frameworks. The CS surveys collected three river attributes (four in winter) and two survey contents that were not collected in the Nation. Using these data, we identified the possibility of predicting bird populations during the winter (Table 3). In other studies, the surface area of water bodies was the most influential covariate explaining bird distribution^48,49^. Similarly, we found that the width of the channel-related surface area of water bodies was positively correlated with bird populations, indicating that channel width is an important factor in bird habitats. In the survey contents, only distance had a positive relationship with bird populations. Although only two of the river attributes and survey contents had significant results, we confirmed that bird communities could be predicted using CS-specific data, indicating that CS was effective. In future studies, to improve data quality, standardised tools should be provided or loaned to participants, along with training them on the usage^24^. In addition, unlike the Nation fixed owing to policy practices, CS allows for flexible adjustments in research. Therefore, if factors that are expected to affect birds, such as floating populations or land use status, are additionally surveyed, they can be utilised not only for predicting the number of individuals but also for conservation strategy purposes.

Our results suggest improvements for future CS monitoring, which can be used as a supplementary dataset for government or professional research. First, provide a systematic training programme. Many CS projects have embedded pretraining or skill tests to select volunteers; these processes have proven to be the most effective approaches for improving results^24^. This study also provided a pre-training program of 1 h, but it was relatively short compared with other CS projects with 4 h or several days of training^50,51^. Therefore, it is necessary to improve the training program by (1) exposing volunteers to images or videos of birds that arrive during the monitoring seasons and (2) conducting additional training for citizens who do not reach the threshold of skill tests for species identification^24,36^. Second, enhanced access to citizen monitoring. Accessibility to participants is an important factor in CS, which has the advantage of collecting vast data across a wider range^52^. With the recent development of the Internet and smartphones, accessibility has become increasingly convenient. Projects with a long history, such as CBC and PFW, which have been conducted since the 1900s, are currently sharing information and recruiting participants online^17,53^. Furthermore, the accessibility of research results must also be considered. Successful CS is not limited to data collection; data management and delivery are also important^54^. Application-based projects such as eBird, iNaturalist, and Korea's Naturing manage the delivery and usage of data by sharing the results of observations in real-time^55,56^. This study also shared information and recruited participants on the internet for increased accessibility; however, the system for managing the collected results remains inadequate. Therefore, it is necessary to establish a systematic online system to enhance accessibility to project participation and results. Third, establish and share specific research methods. Representative bird CS projects, such as CBC (www.audubon.org) and BBS (www.pwrc.usgs.gov/bbs/) provide detailed survey methods for each project. Sharing specific research methods can be one of the most important considerations when proceeding CS, which anyone can participate regardless of knowledge or experience. In this study, the method was explained via online lecture, but the lecture was provided only to participants. Therefore, it is necessary to share information about the methods so that anyone can view them, regardless of their participation status. Finally, get experience through continuous monitoring. The summer monitoring in this study showed that the reliability of the second monitoring period was higher than that of the first. Kelling et al.^35^ showed that bird species identification and detection abilities increased with accumulated experience. In many other CS projects, the data accuracy has a positive relationship with experience^36,57^. Thus, it is necessary to gain the experiences of citizens via continuous monitoring.

## Methods

### Study site

South Korea (33°–38° N, 125°–132° E) located in the middle of the EAAF has a temperate climate with four distinct seasons. The summers are wet and winters dry, with an annual mean temperature of 13.5 °C and an average temperature range of 9.6–18.5 °C. The average annual rainfall is approximately 1240 mm, with more than 60% of the total rainfall recorded during the summer rainy season from June to September due to the East Asian monsoon^58,59^. South Korea has a total area of approximately 96,929 km^2^, of which, 65% (62,684 km^2^) is mountainous, 2% (2042 km^2^) is rivers, 2.6% (2482 km^2^) is coastal, and 1.2% (115 km^2^) is inland (www.kosis.kr). Approximately 40% (120) of the migratory waterbirds in the EAAF use wetlands in South Korea, especially near the West Sea, which accounts for 84% of the coastal wetlands used by approximately 25% (2 million) of the shorebirds in the EAAF (^60^; www.eaaflyway.net).

### Citizen recruitment

Participants were recruited from environmental civic organisations across South Korea, targeting anyone with an interest in avian. All procedures were performed in accordance with Pusan National University Laboratory Safety Management Center (http://labs-safety.pusan.ac.kr) guidelines. All research activities were organised by the Korea Network for Rivers and Watersheds (www.koreariver.or.kr/main/). All participants received ethics education in accordance with Article 29 of the Occupational Safety and Health Act of South Korea (Act No. 18426, 17 August 2021). The privacy of the participants was also protected as no sensitive or personally identifiable information was collected during the study. All participants were provided with information about the purpose and procedures of the study before their involvement, and informed consent was obtained, acknowledging their understanding of the study’s purpose, voluntary participation, and the use and protection of their data. Private and personal information of the participants was protected by removing any and all identifying data from the study parameters. A total of 172 civic organisations, including 801 citizens, participated in this 2-year study. To improve the data quality, the participants underwent training by experienced professionals through online lectures before monitoring. The training session was conducted for approximately 1 h, focusing on learning methods of monitoring, species identification, and field note-taking, to increase the monitoring consistency and reliability of species identification.

### Citizen science monitoring

**Figure 5:**
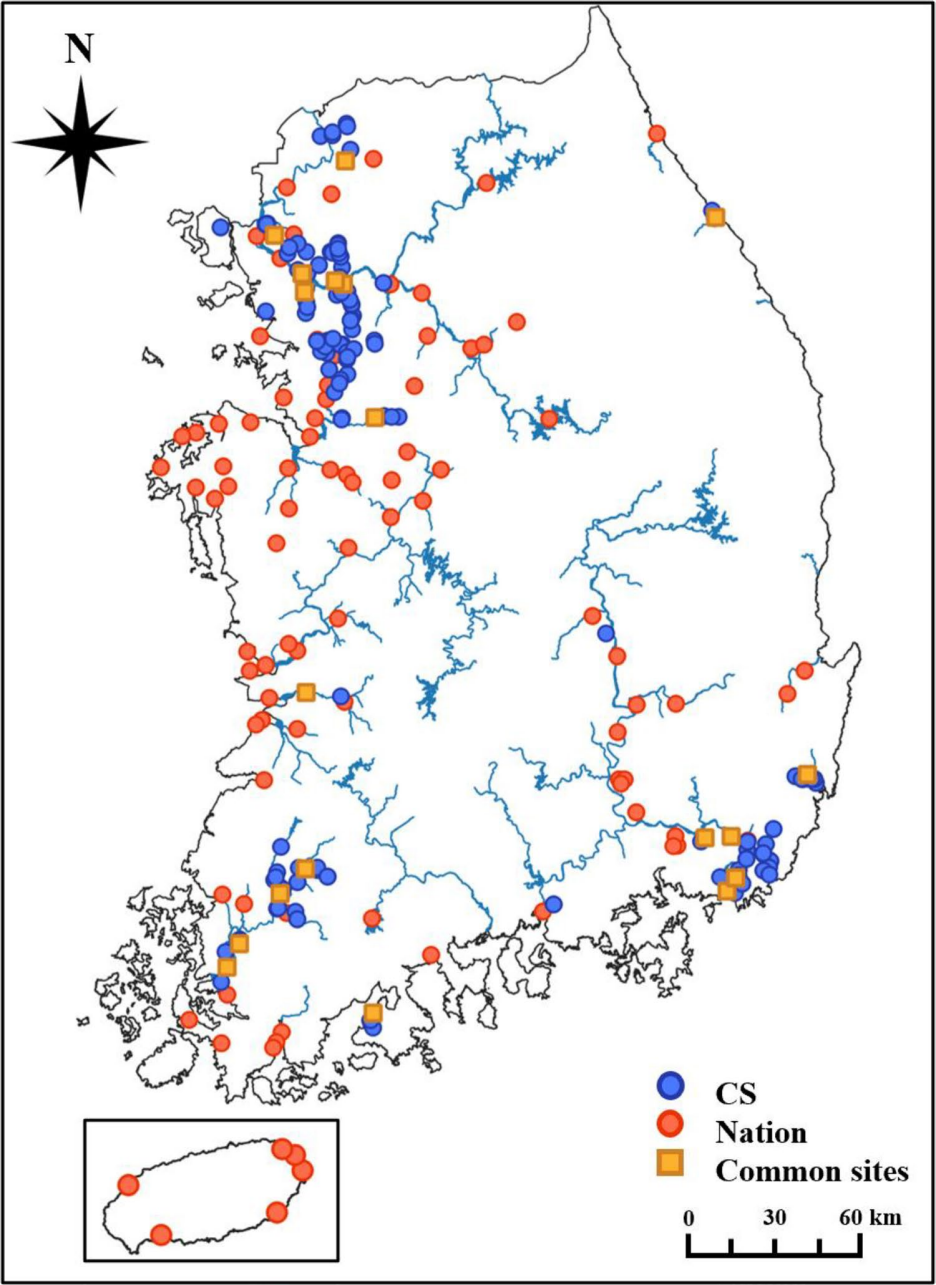
Monitoring sites of citizen science (CS) and national data (Nation). Map created using QGIS ver. 3.22.8 (https://www.qgis.org) with a raster basemap from Ministry of Land, Infrastructure and Transport. Blue circle: CS; Red circle: Nation; Yellow square: common sites of CS and Nation.

CS monitoring was conducted twice a year from 2021 to 2022 to identify the status of migratory birds arriving in the summer and winter (n = 4). All participants were simultaneously monitored to eliminate duplicate counts and minimise time-dependent errors. Monitoring was completed at sites (such as streams, lakes, and reservoirs) inhabited by birds within the operational range of civic organisations, and was carried out at 51 sites in February 2021, 38 sites in June 2021, 60 sites in February 2022, and 42 sites in July 2022 (Fig. 5: The map was created using QGIS ver. 3.22.8 (https://www.qgis.org)). The locations of the survey sites were selected from among participants. For an even distribution of the study sites, overlapping and closely located sites were adjusted in advance.

Bird monitoring was conducted followed a ‘Winter Waterbird Census of Korea’ method. In areas where a species was present in large flocks, the number of individuals was counted in real time with the help of a professional. Species were identified by professionals by shared photographs (e.g. jpg and png), videos (e.g. mp4), and audio recordings (e.g. mp3) on a mobile instant messenger application (KakaoTalk Messenger ver. 9.5.2; Kakao Corp., Seoul, Republic of Korea) and a sharing platform (Naturing ver. 2.1.6; Naturing Inc., Seoul, Republic of Korea) to increase the accuracy of species identification^24^.

To obtain the habitat characteristics, the width of the riparian (m), channel (m), and water depth (cm) were measured, and the freezing rate was investigated during the winter survey. The survey was conducted only with participants equipped with these tools.

### Professional data

To identify the applicability of the data collected by the CS, we used the nationwide ‘Winter Waterbird Census of Korea’ data for Korea from the National Institute of Biological Resources (www.species.nibr.go.kr) for comparison. Observers groups comprising two people as the basic unit moved around the survey area by vehicle, boat, or on foot, recording all species and numbers of individuals observed at the target sites, including the water surface and adjacent wetlands, as well as in neighbouring agricultural areas. Binoculars and telescopes were used as observation equipment, and in cases where identification was impossible due to poor weather conditions or long distances, the species were classified into taxon levels and included only in the number of individuals. During the survey, close communication was maintained with adjacent survey areas to confirm the direction of bird movement and arrival locations, preventing the duplication of individual counts^10,11^.

We organised the population-per-species data of each monitoring session conducted from 19 to 21 February, 2021, and 20 to 22 February, 2022, to minimise timing differences with the CS, and the survey was conducted at 111 and 206 sites (Fig. 5), respectively.

### Data analyses

Benford's law, initially introduced by Newcomb, describes data collected extensively in various fields using mathematical patterns^61^. This law easily verifies complex ecological data and effectively identifies anomalous data. Its use has increased with the growing importance of CS^33,62–64^. We used Benford’s law to assess the CS and Nation data reliability, which was calculated using all counted bird populations during the survey period. Benford’s law proposes that numbers with the first digit of one are observed more often than those starting with two, three, and so on. The probability of appearance of the first digit followed a logarithmic law (Eq. 1):

1 $${P}_{D}={{\text{log}}}_{10}(1+\frac{1}{D})$$

where *P*_*D*_ is the probability of occurrence of the first digit D (D = 1, 2, …, 9)^65^.

To estimate the extent to which the data conform to the theoretical expectations of Benford’s law, we used the chi-square (χ^2^) test of goodness of fit. This test is one of the most common statistical procedures used to assess null hypothesis^66^. This test can be expressed using Eq. (2):

2 $${\upchi }^{2}= {\sum }_{{\text{i}}=1}^{{\text{k}}}\frac{{({O}_{i}- {E}_{i})}^{2}}{{E}_{i}}$$

where *O*_*i*_ is the frequency observed by CS and *E*_*i*_ is the frequency expected from Benford’s law^67^. The p-value was calculated using eight degrees of freedom, so that i = 1, …, 9. The critical values (10, 5, and 1%) for χ^2^ with eight degrees of freedom, were 13.36, 15.51, and 20.09, respectively. Benford’s analysis was performed using the *benford* function in the benford’s analysis package in R ver. 4.2.1 (^68^; R Core Team, 2022).

To determine the effects of the methodology on the diversity assessment, we measured the alpha and beta diversities for all sites. The alpha diversity of CS and Nation data was assessed using two indices: the number of species and the Shannon index at each site. It was assessed using the *diversity* function in the vegan package in R ver. 4.2.1 (^69^; R Core Team, 2022). Beta diversity was measured by community dissimilarity using presence-absence data based on the Sørensen pairwise species dissimilarity (β_SOR_), which measures the proportion of taxa not shared by each site^70^. Dissimilarity analysis based on presence-absence data gives more weight to rare species, considering that common species found at most sites contribute little to between-site differences^71^. We used *beta.multi* functions in the betapart package in R ver. 4.2.1 (^72^; R Core Team, 2022) to calculate the Sørensen dissimilarity. Additionally, we partitioned this dissimilarity additively into turnover (Simpson dissimilarity, β_SIM_), representing the replacement of some species by others, and nestedness (β_SNE_), a measure sensitive to species loss (or gain) components^41,73^. For the diversity analysis, we used only winter data for comparison between CS and Nation. The significance test of the diversity values between CS and Nation was computed using the stats package in R ver. 4.2.1 (R Core Team, 2022).

To identify a difference in the observed tendency of the bird community at common sites depending on the method, we analysed correlations with the number of species and individuals per family and the number of individuals per species, once each for the CS and Nation^74^. We arbitrarily combined data across all samples with CS as the x-axis and Nation as the y-axis and calculated the slope of the line between CS and Nation. The analysis was conducted twice (for all values and for values excluding outliers)^75^. Outliers were detected separately for CS and Nation using the interquartile range^76^. Families or species that appeared as outliers in at least one of the methods were excluded. We conducted a non-parametric correlation test (Spearman’s rho) based on the species observed by both methods using SPSS ver. 26 (IBM Corp. NY, USA, Armonk).

We conducted multiple linear regression analyses to assess the effects of river attributes and survey content on bird abundance. The dependent variable was the total number of individuals. The independent variables used for the river attributes were (1) width of riparian (m), (2) width of channel (m), (3) water depth (m), (4) freezing rate (%), (1) number of citizens, and (2) distance (km). The analysis was conducted separately for summer and winter because freezing rate data were collected only for winter. The data were analysed after normalisation using log transformations. Multiple linear regression analysis was performed using SPSS ver. 26 (IBM Corp. NY, USA, Armonk).

### Supplementary Information

Supplementary Information.

## Data Availability

All data excluding national data are available from the corresponding author on reasonable request. The national data is available from the National Institute of Biological Resources (https://www.nibr.go.kr/).
