# Supplementary material for: Application of citizen science with the nationwide bird census
Source: Sci Rep. 2024 May 6;14:10379. doi: 10.1038/s41598-024-61225-w (PMC11074294; doi:10.1038/s41598-024-61225-w)
Supplement: Supplementary file 1 — Supplementary Information. [file 41598_2024_61225_MOESM1_ESM.docx]

**Application of citizen science with the nationwide bird census**

Yerim Lee^1^, Yuno Do^2^, Maurice Lineman^3^, Gea-Jae Joo^1^, Hyunbin Jo^1,4*^

^1^ Department of Integrated Biological Science, Pusan National University, Busan, Republic of Korea

^2^ Department of Biological Science, Kongju National University, Gongju, Republic of Korea

^3^ RCF Experimental School, Chaoyang District Beijing, P. R.

^4^ Institute for Environment and Energy, Pusan National University, Busan, Republic of Korea

^*^ Corresponding author

Hyunbin Jo: prozeva@pusan.ac.kr

Supplementary Table S1. Frequency table of the first digit from citizen science and national data by survey period compared with Benford’s law. BL, Benford’s law; CS: Citizen Science; W, winter monitoring; S, summer monitoring.

| Digits | BL | CS | | | | | | | | Nation | | | |
| --- | --- | --- | --- | --- | --- | --- | --- | --- | --- | --- | --- | --- | --- |
|  |  | 2021W | | 2021S | | 2022W | | 2022S | | 2021 | | 2022 | |
|  |  | Observed | Data set | Observed | Data set | Observed | Data set | Observed | Data set | Observed | Data set | Observed | Data set |
| 1 | 30.10% | 304 | 33.67% | 172 | 35.10% | 404 | 34.38% | 180 | 34.95% | 1414 | 30.91% | 2834 | 32.96% |
| 2 | 17.61% | 167 | 18.49% | 102 | 20.82% | 233 | 19.83% | 94 | 18.25% | 993 | 21.70% | 1804 | 20.98% |
| 3 | 12.49% | 116 | 12.85% | 61 | 12.45% | 146 | 12.43% | 60 | 11.65% | 564 | 12.33% | 1056 | 12.28% |
| 4 | 9.69% | 83 | 9.19% | 44 | 8.98% | 82 | 6.98% | 48 | 9.32% | 449 | 9.81% | 787 | 9.15% |
| 5 | 7.92% | 73 | 8.08% | 41 | 8.37% | 89 | 7.57% | 35 | 6.80% | 331 | 7.23% | 622 | 7.23% |
| 6 | 6.70% | 72 | 7.97% | 16 | 3.27% | 76 | 6.47% | 35 | 6.80% | 277 | 6.05% | 513 | 5.97% |
| 7 | 5.80% | 42 | 4.65% | 23 | 4.69% | 59 | 5.02% | 29 | 5.63% | 195 | 4.26% | 391 | 4.55% |
| 8 | 5.12% | 31 | 3.43% | 22 | 4.49% | 58 | 4.94% | 27 | 5.24% | 206 | 4.50% | 358 | 4.16% |
| 9 | 4.58% | 15 | 1.66% | 9 | 1.84% | 28 | 2.38% | 7 | 1.36% | 146 | 3.19% | 233 | 2.71% |
| Chi-Square | | 30.5801** | | 25.3604** | | 33.2766** | | 17.0215* | | 91.3903** | | 197.4877** | |

Note: * indicates 95% and ** indicates 99%, significantly different from Benford’s.
